# Mortality Risk Assessment Using the REVEAL 2.0 Score in Pulmonary Hypertension Secondary to Left Heart Disease

**DOI:** 10.21203/rs.3.rs-4474171/v1

**Published:** 2024-06-25

**Authors:** Demetrio Sharp-Dimitri, Mahyar Pourriahi, Christine Zhou, Roman Jandarov, Dana Kay, Arun Jose, Jennifer Cook, Jean Elwing, Jose Gomez-Arroyo

**Affiliations:** University of Cincinnati; University of Cincinnati; University of Cincinnati; University of Cincinnati; University of Cincinnati; University of Cincinnati; University of Cincinnati; University of Cincinnati; University of Cincinnati

**Keywords:** Pulmonary Hypertension, Heart Failure, Heart Failure with Reduced Ejection Fraction, Heart Failure with Preserved Ejection Fraction, Risk Stratification, Mortality Prediction

## Abstract

**Background:**

Pulmonary hypertension (PH) frequently complicates the course of patients with left heart disease (PH-LHD) and is associated with worse clinical outcomes. Mortality calculators for PH-LHD are lacking, and it is unclear whether any risk prediction tools originally derived from other forms of PH can accurately predict outcomes in patients with PH-LHD.

**Methods:**

We retrospectively analyzed data from 161 patients diagnosed with PH-LHD referred to our pulmonary hypertension center from 2016 to 2022. We calculated the Registry to Evaluate Early and Long-Term PAH Disease Management (REVEAL 2.0) risk score and categorized patients as low, intermediate, or high-risk. We assessed survival at 1 and 3 years using Kaplan-Meier and Cox proportional hazards, as well as classification performance using a concordance index.

**Results:**

At the first outpatient visit, 15% of patients were stratified as low-risk, 27% as intermediate, and 57% as high-risk. Cumulative 1-year survival rates were 100%, 94%, and 91% for the low, intermediate, and high-risk strata, respectively. Cumulative 3-year survival rates were 96%, 89%, and 70% for the low, intermediate, and high-risk strata, respectively. We found no difference in outcomes at 1 year between risk groups. High-risk patients had an increased risk of death at 3 years using REVEAL 2.0 (HR 5.32, p < 0.001). However, while REVEAL 2.0 accurately discriminated high-risk patients, the hazard ratio was not statistically different between patients classified as intermediate-risk compared to low-risk.

**Conclusion:**

REVEAL 2.0 accurately predicted 3-year survival in PH-LHD patients with high-risk features. However, the mortality risk between patients classified as intermediate-risk was not different from the low-risk stratum, suggesting inaccurate classification for this group of patients.

## Introduction

Pulmonary hypertension (PH) associated with left heart disease (LHD), defined by a mean pulmonary arterial pressure (mPAP) > 20 mmHg and a pulmonary arterial wedge pressure (PAWP) or left ventricular-end diastolic pressure (LVEDP) > 15 mm Hg^1^, is the most common type of PH, with an estimated prevalence ranging between 40%−80% in all patients with heart failure (HF)^2 3^. In addition to its high prevalence, numerous studies have shown an inverse relationship between PH and survival in patients with HF, particularly when associated with right ventricular (RV) dysfunction (i.e. biventricular dysfunction)^4,5 6^. Interestingly, despite its high frequency and impact on disease progression, risk prediction tools are lacking in this PH subtype.

Several risk prediction scores have been derived and validated to estimate the probability of death in patients with pulmonary arterial hypertension (PAH), including the Registry to Evaluate Early and Long-term PAH Disease Management Registry (REVEAL) and its derivatives (REVEAL 2.0 and REVEAL-Lite^7^). However, whether any of these tools perform with precision in a different population of patients without PAH (Group 1 PH), specifically PH-LHD, remains to be investigated. Here we aimed to assess the prognostic utility of the REVEAL 2.0 model in predicting survival in a cohort of patients with PH-LHD at 1 and 3 years.

## Methods

### Definition of Study Population and Clinical Variables

We retrospectively analyzed data from patients referred to the Pulmonary Hypertension Clinic at the University of Cincinnati from 2016–2022. Patients were diagnosed with PH-LHD using the 2022 ESC/ERS pulmonary hypertension guidelines^8^, with the following hemodynamic thresholds, obtained by right heart catheterization (RHC) at an optimized euvolemic state: 1) mPAP > 20 mmHg at rest and 2) PAWP > 15 mmHg^9^. According to ERS/ESC guidelines, isolated post-capillary pulmonary hypertension (ipcPH) was defined as mPAP > 20 mmHg, PAWP > 15 mmHg and a PVR 2 ≤ Wood Units (WU). Combined pre and post-capillary pulmonary hypertension (cpcPH) was defined as mPAP > 20 mmHg, PAWP > 15 mmHg and a PVR > 2 WU. All RHC tracings were reviewed by a pulmonary hypertension specialist (J.M.E, J.G-A) to confirm the validity of the PCWP tracings. HF with preserved ejection fraction (HFpEF) and HF with reduced ejection fraction (HFrEF) were defined based on signs and symptoms consistent with HF failure and a left ventricular ejection fraction (LVEF) estimated by echocardiography of ≥ 50% or LVEF ≤ 40%, respectively.^10^ Co-morbidities such as hypertension, dyslipidemia, diabetes, COPD and OSA chronic kidney disease were defined as per ICD-10 codes in the electronical medical record system. Coronary artery disease was defined as a combination of ICD-10 or evidence by left heart catheterization or CT-based imaging. Obesity was defined as having a BMI higher or equal to 30. Atrial fibrillation, as well as other electrocardiographic abnormalities were determined by ICD-10 code and independent EKG review (D.S.D).

### Risk Stratification using the REVEAL 2.0 Model

As previously reported, the REVEAL 2.0 risk score is comprised of thirteen variables^11^ (Online *Supplemental Material*). Each variable contributes to a weighted risk; a minimum of 7 variables is required to generate an accurate score. The REVEAL 2.0 score assigns patients to a risk category based on the weighted score as follows: Low-risk (score of ≤ 6), intermediate-risk (score of 7 or 8), and high-risk (score of ≥ 9. To assign patients a risk score, we collected demographic data, clinical characteristics, laboratory, and hemodynamic data closest to the first outpatient clinic visit (OV-1) after a diagnosis of PH-LHD was established. Specifically, echocardiographic, relevant laboratory (natriuretic peptide levels [BNP and NTproBNP], and renal function tests), and clinical (demographics, vital signs, New York Heart Association-World Health Organization Functional Class (NYHA/WHO-FC), six-minute walk distance [6MWD]) data collected within 3 months of the OV-1 was used, and RHC hemodynamic data within 1 year of the OV-1 was used for calculation of each subjects’ initial REVEAL 2.0 risk score. The University of Cincinnati Institutional Review Board approved this study protocol and all related activities (IRB Protocol Number 2020 − 0466).

### Statistical Methods

Categorical variables are reported as percentages, while continuous variables are reported as median and interquartile range (IQR). Between-group differences were compared by Wilcoxon rank-sum test, or *chi*^2^ tests as appropriate. Spearman’s coefficient was used to calculate correlations. The concordance index was calculated to evaluate the performance of the REVEAL score in classifying survivors versus non-survivors at 1 and 3 years from the OV-1. Kaplan-Meier curves were used to compare mortality at 3 years following OV-1. Cox proportional hazard models were used to examine the relationships between risk stratification and 3-year survival. Continuous variables (BNP, 6MWD, and pulmonary vascular resistance [PVR] were standardized to z-scores to allow comparisons of odds ratios based upon a one standard deviation change in a given variable. Cox proportional hazards models were adjusted for relevant demographic data in a multivariable fashion. All tests were two-sided, and statistical significance was set at *p < 0.05*. Analysis was performed using IBM SPSS version 28.0 (Armonk, NY) and R Studio (R version 4.2.3), using the *survival, Epi* and *ROC* packages.

## Results

### Cohort Demographics, Clinical Characteristics, and Hemodynamic Data at the First Outpatient Visit

We included a total of 161 individuals who met criteria for pulmonary hypertension due to left heart disease (PH-LHD), all of whom had comprehensive clinical and hemodynamic data available. *Table 1* presents a summary of the baseline demographic characteristics, laboratory findings, and hemodynamic parameters at the initial outpatient visit (OV-1). The median age of the participants was 66 years (IQR 18 years). The cohort was predominantly female sex (68%). HFpEF was the most frequent subtype of HF (85.1% [n = 137]). In the subset of patients with HFrEF, the median left ventricular ejection fraction (LVEF) by echocardiography was 33% (IRQ 17.5%). Most people were categorized as a NYHA/WHO FC III (77.6% [n = 125]) and had a 6MWD of 244 meters (IQR 169.7 meters). Only a minority of patients (3.1% [n = 5]) presented with a NYHA/WHO FC IV. Over half of patients had coronary artery disease (53.4% [n = 86]), out of which a majority had undergone some form (surgical or percutaneous) of coronary revascularization (66.3% [n = 57]). Comorbid obesity (72%, [n = 117]), hypertension (98.8% [n = 159]), and hyperlipidemia (84.5% [n = 136]) were highly prevalent in the cohort. Obstructive sleep apnea (84.5% [n = 136]) and chronic obstructive lung disease (COPD) (33% [n = 53]) were the two most frequent pulmonary co-morbidities. The median forced expiratory volume percentage (FEV1%) predicted in patients carrying a diagnosis of COPD was 61% (IQR 25%). Eighteen patients (11%) had an FEV1% predicted below 50%.

Almost half of the study sample (47.8% [n = 77]) had reduced eGFR (< 90 mL/min/1.73m2) suggesting chronic kidney disease (CKD), with an overall median eGFR of 60.0 mL/min/1.73m2 (IQR 39 mL/min/1.73m2). As expected, loop diuretics were highly prevalent among all patients (81.4% [n = 131]). Other frequently used medications included beta-adrenergic receptor antagonists (52.2% [n = 84]), and mineralocorticoid receptor antagonists (MRA) (24.8% [N = 40]).

Patients with HFpEF had a higher TAPSE on echocardiogram (2.0 cm [IQR, 0.85] vs. 1.7 [IQR, 0.5], p < 0.001) and achieved a longer 6MWD (257 meters [IQR, 147] vs 155 meters [IQR, 166.0], p < 0.01) compared to patients with HFrEF. Patients with HFrEF more frequently exhibited higher median NT-ProBNP (4962.1 pg/ml [IQR 5549.0] vs 581 [IQR 1787.0], p < 0.001), and more often exhibited electrocardiographic abnormalities, including a widened QRS complex and bundle branch blocks (**Table 1**).

Regarding cardiopulmonary hemodynamics for the entire cohort, the median right atrial pressure (RAP) was 12.0 mmHg (IQR, 7.0), mPAP was 35.0 mmHg (IQR, 15.0), PCWP was 20.0 mmHg (IQR, 7.0) and pulmonary venous resistance (PVR) was 2.5 Wood units (IQR, 2.6). Median cardiac output (CO) and cardiac index (CI) by Indirect Fick’s method^12^ were 5.8 L/min (IQR, 2.7) and 2.73 L/min/m2 (IQR, 1.2), respectively. Patients with HFrEF had a higher PVR (4.1 W.U [IRQ 3.25] vs 2.0 W.U [IQR 2.30], p < 0.001), higher mPAP (41.5 mmHg [IQR 17.5] vs 35 mmHg [IQR 15.0], p < 0.05), and lower cardiac output (4.22 L/min [IQR 2.1] vs 6.4 L/min [IQR 2.75], p < 0.001) compared to patients with HFpEF. Sixty six percent of subjects (N = 104) met the hemodynamic Definition of cpcPH. There was no difference in the proportion of patients with cpcPH between HFrEF or HFpEF (62%, [N = 15] vs 67%, [N = 89], p = 0.674).

#### Differences in Demographics, Clinical Characteristics, Echocardiographic and Hemodynamic Data Between REVEAL Risk Groups.

REVEAL 2.0 scores for the entire cohort at OV-1 are shown in **Table 2.** Roughly half the cohort (N = 84, 52.2%) were classified as high-risk, and almost one-third (N = 45, 28%) were intermediate risk. High-risk stratum patients tended to be older with a lower BMI and more frequently had comorbid medical conditions including atrial fibrillation (64.3%, N = 54), coronary artery disease (64.3%, N = 54), diabetes mellitus (64.3%, N = 54), HFrEF (25%, N = 21), and CKD (65.5%, N = 55) (**Table 3**).

**Figure 1:**
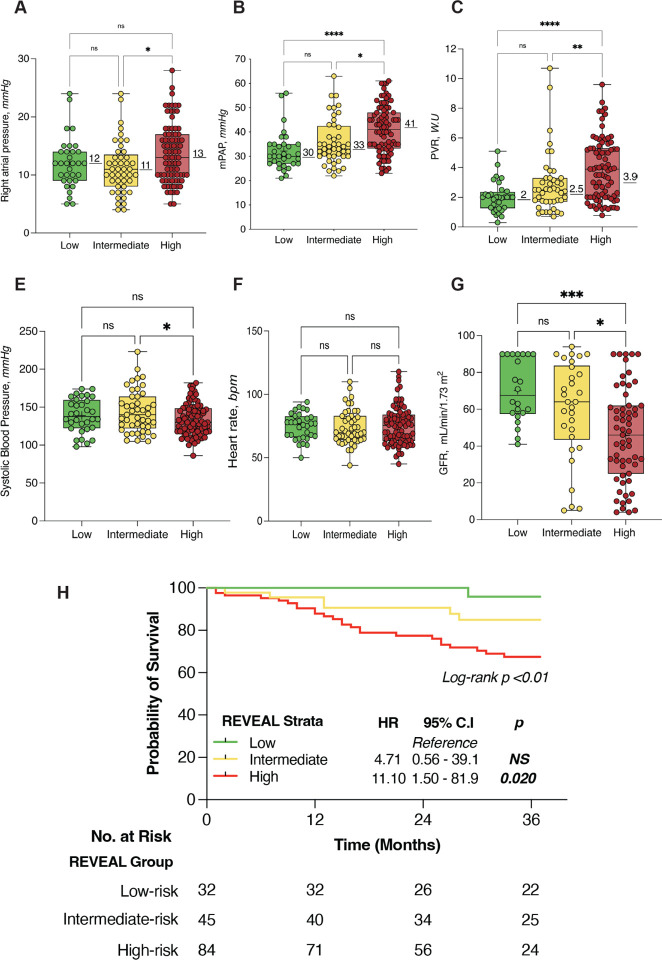
Box and whisker plots showing the distribution of hemodynamic data (**panels A-C**), as well as other key components of the REVEAL 2.0 score (**panels E-G**) between patients categorize as low (green), intermediate (yellow), and high (red) risk strata. mPAP= Mean Pulmonary Arterial Pressure; TPG= Transpulmonary Gradient; PVR= Pulmonary Vascular Resistance. **Panel H**: Kaplan-Meier estimates of cumulative 3-year survival from the first outpatient visit. Cox-proportional hazard ratios (HR) for intermediate and high-risk strata categories compared to the reference (low-risk) are shown. *= P<0.05; **= P<0.01; ***= P<0.001; NS= Not Statistically Significant.

Unsurprisingly, high-risk patients exhibited a worse hemodynamic profile compared to low-risk strata. As illustrated in Fig. 1, mPAP, PCWP, and PVR were significantly higher in high-risk compared to low-risk patients. However, no survival differences were found between patients in low versus intermittent-risk strata.

### Survival Analysis

Of the initial 161 patients in our study, 10 patients died within 1-year of OV-1, and 32 patients died within a 3-year period (**Table 4**). Survivors had a lower frequency of comorbid medical conditions at OV-1, including coronary artery disease (47.3% vs. 78.1% [p = 0.002]), ischemic cardiomyopathy (24.8% vs. 43.8% [p = 0.034]), and atrial fibrillation (45.7% vs. 68.8% [p = 0.002) as compared to non-survivors. There was an equal proportion of patients with HFrEF and HFpEF between groups. Similarly, we found the same frequency of cpcPH/ipcPH between survivors and non-survivors. Survivors had a higher TAPSE (2.1 [IQR 0.9] vs 1.8 [IQR 0.4], p < 0.05) and lower median NT-ProBNP (581 [IQR, 1379] vs 3181.0 [IQR, 7636], [p < 0.001]) compared to non-survivors. Consistently, logistic regression analysis showed a significant association between TAPSE and risk of death (OR 0.42; 95% C.I, 0.189–0.932, [*p < 0.05]*). However, this relationship was dependent on LVEF%.

We found a significant difference in mPAP ((35 mmHg [IQR 14.5] vs. 41 mmHg [IQR 17.8], p = 0.004) and PVR (2.4 Wood units [IQR 2.25 ] vs. 3.9 Wood units [IQR 3.5], p = 0.027) between survivors and non-survivors. However, there were no significant differences in RAP, PCWP or CO/CI between groups (**Table 2**). Logistic regression analysis demonstrated a 49% increase in the risk of death by every 1 SD (2.05 WU) increase in PVR (OR 1.497, 95% C.I, 1.037–2.161, p < 0.05]). Similarly, we found an 82% increase in the risk of death by every 1 SD (10 mmHg) increase in mPAP (OR 1.823, 95% C.I, 1.23–2.69, [P < 0.001]). Lastly, although there was a trend towards better outcomes, having ipcPH was not associated with statistically lower odds of death compared to cpcPH (OR 0.764; 95% CI 0.339–1.722, *p = 0.516*).

The cumulative survival between low, intermediate, and high-risk risk groups at 1-year were 100%, 94% and 91%. At 3-years, the survival estimates were 96%, 89% and 70%, respectively (Fig. 1H). Cox-proportional hazard analysis showed a 43% increase in mortality risk at 3-years by every 1 point increase in the REVEAL 2.0 score (HR 1.43, 95% C.I, 1.24–1.647, [p < 0.001]). Consistently, we found an 11-fold increase in the risk of death for high-risk stratum (HR 11.10; 95% CI, 1.50–81.9, [p < 0.05]) compared to the reference (low-risk stratum). Importantly, whereas we found a trends towards higher mortality risk in patients classified as intermediate-risk, it did not reach the significant threshold (HR 4.71; 95% CI, 0.56–39, [p = 0.151]). The C-index to classify survivors at 3 years was 0.680.

When analyzing specific variables included in the REVEAL 2.0 score we found that DLCO percent predicted < 40% (HR 2.32, 95% C.I, 1.043–5.167, [p < 0.05]) and right atrial pressure > 20 mmHg (HR 2.218, 95% C.I, 1.182–4.160, [p < 0.05]) were significantly associated with increased mortality. Similarly, we found a 30% increase in the risk of death per one standard deviation change in Z-standardized BNP/NT-Pro BNP (HR 1.302 per 1 SD, 95% C.I 1.052–1.611, [p < 0.05]), 6MWD (HR 0.604 per 1 SD, 95% C.I 0.377–0.971, [p < 0.05]) and PVR (HR 1.384 per 1 SD, 95% C.I 1.030–1.860, [p < 0.05]). However, age > 60 years (HR 1.392, 95% C.I, 0.966–2.006, *p = 0.076*), NYHA functional class (HR 3.03, 95% C.I, 0.981–9.392, *p = 0.054*), hospitalizations within 6-months (HR 1.102, 95% C.I, 0.544–2.232, *p = 0.074*), systolic blood pressure < 110 mmHg (HR 1.553, 95% C.I, 0.639–3.774, *p = 0.331*), heart rate > 96 (HR 2.57, 95% C.I, 0.990–6.691, *p = 0.052*), pericardial effusion (HR 0.852, 95% C.I, 0.351–2.071, p = 0.724) and GFR < 60 mL/min/1.73 m2 (HR 1.144, 95% C.I, 0.572–2.287, *p = 0.704*) did not reach statistical significance.

Figure 1E–G illustrates the distribution of values for systolic blood pressure, heart rate and GFR between REVEAL risk groups. Notably, there were no differences in systolic blood pressure, heart rate of GFR between low and intermediate-risk groups, which could explain why the mortality risk intermediate-risk stratum was not statistically different from the low-risk category. Finally, multivariable Cox proportional hazard analysis including all REVEAL 2.0 variables demonstrated that only Z-standardized BNP/NT-ProBNP (HR 1.56, 95% C.I, 1.05–2.31, [p < 0.05]) was independently associated with higher mortality (**Table 5**).

## Discussion

In this single-center retrospective analysis, we found that in adults with PH-LHD, the REVEAL 2.0 score accurately separated patients at the highest risk of death at 3 years from the first outpatient visit. However, patients classified as intermediate-risk did not show a significant difference in survival compared to the low-risk strata. The c-index for the REVEAL 2.0 score was 0.68, which is below the threshold for good discrimination and lower than what has been reported for patients with PAH (0.73, 95% CI, 0.71–0.75^7^). We observed a significant overlap in the distribution of values for systolic blood pressure, heart rate, and GFR between low and intermediate-risk groups, which may affect the overall classification performance. Like published data for PAH, BNP/NT-pro BNP was the strongest biomarker driving risk stratification and outcomes in our sample of patients with PH-LHD. Collectively, our results suggest that the REVEAL 2.0 risk score may have prognostic value in predicting survival in PH-LHD patients, however also underscores certain limitations that could help improve classification performance.

Despite a high prevalence and significant effect on morbidity and mortality, PH in the context of HF tends to be underestimated^13^. Indeed, most clinically relevant mortality calculators for HF do not consider the presence of PH or right ventricular dysfunction as co-variates when assessing risk^14 15^. Prognostic equations and risk prediction models originally developed to predict mortality in PAH have increasingly become an important tool for the management of these patients. However, virtually all calculators were derived from PAH registries and their performance in other types of PH, including PH-ILD remains largely undetermined. Our data suggest the REVEAL 2.0 can accurately discriminate PH-LHD patients at high-risk of death, but it may not accurately distinguish patients with intermediate-risk features. We believe that the significant overlap in the distribution of values between groups for key variables (i.e. variables selected for abridged versions of the score) such as systolic blood pressure, heart rate and GFR plays a significant role in the score’s performance. Indeed, we did not find a difference in any of these 3 parameters between patients stratified as low versus intermediate-risk. Similarly, we did not find a significant difference in these 3 parameters between survivors and non-survivors (data not shown). In contrast to patients with PAH, where low systolic blood pressure is a biomarker of progressive right ventricular dysfunction, there is a significant amount of evidence to support the beneficial effect of reducing systemic vascular resistance in patients with left heart disease, in order to improve forward flow^10^. In addition, more than 50% of our cohort was receiving beta-blocker therapy which likely influenced heart rate. In a similar way, as shown in Fig. 1, we found a broad distribution of hemodynamic values that resulted in significant overlap between low and intermediate-risk patients, suggesting that these parameters are not different enough to differentiate patients with more severe disease, as it would be the case for PAH. Whether or not these clinical or hemodynamic parameters ought to be exchanged or the thresholds modified to reflect a decompensated state in a PH-LHD patient population warrants further investigation.

In contrast, we found that BNP/NT-pro BNP was the only parameters from the REVEAL 2.0 score that independently predicted higher mortality. This is not surprising given that BNP has repeatedly been shown to predict outcomes in patients with left-sided heart failure^16^ and in PAH^17^. Similar findings were reported when the REVEAL Lite 2 score was reported^7^, where Benza and collaborators showed the most highly predictive parameter was BNP/NT-proBNP.

One interesting finding was that, although numerically higher, we did not see a statistical difference in outcomes between patients with ipcPH and cpcPH. This finding may have been influenced by our sample size and will require further investigation. However, Nasri and collaborators^18^, reported similar results in a larger sample of patients with heart failure. We found that elevated mPAP was strongly associated with worse outcomes, irrespective of a diagnosis of ipcPH/cpcPH. Similar findings have been reported by Titz, Ulrich and collaborators^19^ where in their cohort of patients with cpcPH, they found a 58% increase in risk of death when the mPAP was higher than 46 mmHg. Surprisingly, they did not find a relationship between elevated PVR (> 5.8 Wood units) and mortality. Titz *et al*. also failed to find an association between age > 68 yrs and mortality. Compared to patients with PAH, a most common form of HF (i.e., HFpEF]) is almost exclusively a disorder of persons aged 60 yrs or older^20^, and it has been estimated that post-capillary PH is most frequent in HF patients older than 65 yrs^21^. Thus, it is likely that age > 65 years is a parameter without utility for risk stratification in patients with PH-LHD.

In our cohort, a striking majority of patients were classified as high-risk on their first outpatient visit. Most importantly, the survival rates at 3-years were remarkably similar to patients with PAH receiving pharmacotherapies^7^, which is comparable to data published by others^19 21^. We believe this result is likely multifactorial and reflects our incomplete understanding of the mechanisms driving disease progression in patients with PH-LHD, including 1) the effect of biventricular (RV and LV) failure, 2) the impact of highly prevalent co-comorbidities in HF such as coronary artery disease, conduction abnormalities and 3) the lack of specific pharmacotherapy and the effect of beta-blockers, which are frequently avoided in patients with precapillary PH. It is essential to mention that non-survivors exhibited lower TAPSE, highlighting the contribution of RV dysfunction in the prognosis of these patients, even when the absolute difference in TAPSE was not large (average difference 0.198 cm between survivors and non-survivors). Similar results have been shown by Melenovsky *et al*.^22^ in patients with HFpEF with and without concomitant right ventricular dysfunction where even a 15% difference in RV fractional area change was associated (45% vs 30%) with worse outcomes^22^.

### Limitations

Our study had both strengths and limitations. On the one hand, the single center cohort, limited sample size, cross-sectional design, and retrospective nature of our study certainly limits the interpretation of the causality of associations and mechanistic relationships found in this group of patients. Selection bias may have been present, given that only certain patients with HF are referred to a pulmonary hypertension clinic and thus, our results may not be generalizable to a broader population of patients with PH-LHD. However, one important strength of our study was data completion and deep phenotypic characterization.

## Conclusions

The REVEAL 2.0 risk assessment score accurately predicted 3-year survival in PH-LHD patients with high-risk features. However, the mortality risk between patients classified as intermediate-risk was not different from the low-risk stratum, suggesting inaccurate classification for this group of patients. Our findings underscore a significant overlap of clinical and hemodynamic features between intermediate and low-risk patients, which may play a role in classification performance. Further studies are warranted to modify the existing PAH scores to better stratify and estimate mortality in patients with PH-LHD.

## Availability of data and materials:

Data availability is ensured and can be facilitated upon a formal request from the investigator to the corresponding author.

## Abbreviations

PH: Pulmonary Hypertension
HF: Heart Failure
HFrEF: Heart Failure with Reduced Ejection Fraction
HFpEF: Heart Failure with Preserved Ejection Fraction
PH-LHD: Pulmonary Hypertension Associated with Left Heart Diseases
PAH: Pulmonary Arterial Hypertension
MPAP: Mean Pulmonary Artery Pressure
PAWP: Pulmonary Arterial Wedge Pressure
LVEDP: Left Ventricular-End Diastolic Pressure
LVEF: Left Ventricular Ejection Fraction
PVR: Pulmonary Vascular Resistance
WU: Woods Unit
ipcPH: Isolated Post-Capillary Pulmonary Hypertension
cpcPH: Combined Pre- And Post-Capillary Pulmonary Hypertension
NYHA/WHO-FC: New York Heart Association-World Health Organization Functional Class
REVEAL: Registry to Evaluate Early and Long-term PAH Disease Management Registry
OV-1: First Outpatient Clinic Visit
OV-12: Outpatient Visit at 12–18 months of the first clinic visit
OV-36: Outpatient Visit at 36 months of the first clinic visit
FEV1%: Forced Expiratory Volume Percentage Predicted

## References

[1] Vachiéry J, Tedford RJ, Rosenkranz S, Palazzini M, Lang I, Guazzi M, Coghlan G, Chazova I, De Marco T. Pulmonary hypertension due to left heart disease, Eur Respir J 2019;53. 10.1183/13993003.01897-2018 PMC6351334 30545974

[2] Guazzi M, Ghio S, Adir Y. Pulmonary Hypertension in HFpEF and HFrEF: JACC Review Topic of the Week, J Am Coll Cardiol 2020;76:1102–1111. 32854845 10.1016/j.jacc.2020.06.069

[3] Lin Y, Pang L, Huang S, Shen J, Wu W, Tang F, Su W, Zhu X, Sun J, Quan R, Yang T, Han H, He J. The prevalence and survival of pulmonary hypertension due to left heart failure: A retrospective analysis of a multicenter prospective cohort study, Frontiers in Cardiovascular Medicine 2022;9. 10.3389/fcvm.2022.908215 PMC9378855 35983183

[4] Damy T, Goode KM, Kallvikbacka-Bennett A, Lewinter C, Hobkirk J, Nikitin NP, Dubois-Randé J, Hittinger L, Clark AL, Cleland JGF. Determinants and prognostic value of pulmonary arterial pressure in patients with chronic heart failure, European Heart Journal 2010;31:2280–2290. 20693169 10.1093/eurheartj/ehq245

[5] Caravita S, Faini A, Carolino D’Araujo S, Dewachter C, Chomette L, Bondue A, Naeije R, Parati G, Vachiéry J. Clinical phenotypes and outcomes of pulmonary hypertension due to left heart disease: Role of the pre-capillary component, PLoS One 2018;13. 10.1371/journal.pone.0199164 PMC6007912 29920539

[6] Guazzi M, Ghio S, Adir Y. Pulmonary Hypertension in HFpEF and HFrEF: JACC Review Topic of the Week, Journal of the American College of Cardiology 2020;76:1102–1111. 32854845 10.1016/j.jacc.2020.06.069

[7] Benza RL, Kanwar MK, Raina A, Scott JV, Zhao CL, Selej M, Elliott CG, Farber HW. Development and Validation of an Abridged Version of the REVEAL 2.0 Risk Score Calculator, REVEAL Lite 2, for Use in Patients With Pulmonary Arterial Hypertension, Chest 2021;159:337–346. 32882243 10.1016/j.chest.2020.08.2069 PMC7462639

[8] Humbert Marc, Kovacs Gabor, Hoeper Marius M., Badagliacca Roberto, Berger Rolf M.F., Brida Margarita, Carlsen Jørn, Coats Andrew J.S., Escribano-Subias Pilar, Ferrari Pisana, Ferreira Diogenes S., Ghofrani Hossein Ardeschir, Giannakoulas George, Kiely David G., Mayer Eckhard, Meszaros Gergely, Nagavci Blin, Olsson Karen M., Pepke-Zaba Joanna, Quint Jennifer K., Rådegran Göran, Simonneau Gerald, Sitbon Olivier, Tonia Thomy, Toshner Mark, Vachiery Jean-Luc, Noordegraaf Anton Vonk, Delcroix Marion, Rosenkranz Stephan, the ESC/ERS Scientific Document Group. 2022 ESC/ERS Guidelines for the diagnosis and treatment of pulmonary hypertension, Eur Respir J 2022:2200879. 10.1183/13993003.00879-2022 36028254

[9] Paulus WJ, Tschöpe C. A Novel Paradigm for Heart Failure With Preserved Ejection Fraction: Comorbidities Drive Myocardial Dysfunction and Remodeling Through Coronary Microvascular Endothelial Inflammation, Journal of the American College of Cardiology 2013;62:263–271. 23684677 10.1016/j.jacc.2013.02.092

[10] Writing Committee Members, ACC/AHA Joint Committee Members. 2022 AHA/ACC/HFSA Guideline for the Management of Heart Failure, J Card Fail 2022;28:e1–e167. 10.1016/j.cardfail.2022.02.010 35378257

[11] Benza RL, Gomberg-Maitland M, Elliott CG, Farber HW, Foreman AJ, Frost AE, McGoon MD, Pasta DJ, Selej M, Burger CD, Frantz RP. Predicting Survival in Patients With Pulmonary Arterial Hypertension: The REVEAL Risk Score Calculator 2.0 and Comparison With ESC/ERS-Based Risk Assessment Strategies, Chest 2019;156:323–337. 30772387 10.1016/j.chest.2019.02.004

[12] de Boode W, Osypka M, Soleymani S, Noori S. Chapter 14 - Assessment of Cardiac Output in Neonates: Techniques Using the Fick Principle, Indicator Dilution Technology, Doppler Ultrasound, Thoracic Electrical Impedance, and Arterial Pulse Contour Analysis. In: Seri I, Kluckow M, eds. Hemodynamics and Cardiology (Third Edition). Elsevier: Philadelphia, 2019:237–263.

[13] Rosenkranz S, Gibbs JSR, Wachter R, De Marco T, Vonk-Noordegraaf A, Vachiéry J. Left ventricular heart failure and pulmonary hypertension, Eur Heart J 2016;37:942–954. 26508169 10.1093/eurheartj/ehv512 PMC4800173

[14] Levy WC, Mozaffarian D, Linker DT, Sutradhar SC, Anker SD, Cropp AB, Anand I, Maggioni A, Burton P, Sullivan MD, Pitt B, Poole-Wilson PA, Mann DL, Packer M. The Seattle Heart Failure Model: prediction of survival in heart failure, Circulation 2006;113:1424–1433. 16534009 10.1161/CIRCULATIONAHA.105.584102

[15] Crespo-Leiro MG, Anker SD, Maggioni AP, Coats AJ, Filippatos G, Ruschitzka F, Ferrari R, Piepoli MF, Delgado Jimenez JF, Metra M, Fonseca C, Hradec J, Amir O, Logeart D, Dahlström U, Merkely B, Drozdz J, Goncalvesova E, Hassanein M, Chioncel O, Lainscak M, Seferovic PM, Tousoulis D, Kavoliuniene A, Fruhwald F, Fazlibegovic E, Temizhan A, Gatzov P, Erglis A, Laroche C, Mebazaa A. European Society of Cardiology Heart Failure Long-Term Registry (ESC-HF-LT): 1-year follow-up outcomes and differences across regions, Eur J Heart Fail 2016;18:613–625. 27324686 10.1002/ejhf.566

[16] York MK, Gupta DK, Reynolds CF, Farber-Eger E, Wells QS, Bachmann KN, Xu M, Harrell FEJ, Wang TJ. B-Type Natriuretic Peptide Levels and Mortality in Patients With and Without Heart Failure, J Am Coll Cardiol 2018;71:2079–2088. 29747827 10.1016/j.jacc.2018.02.071 PMC5951190

[17] Benza RL, Gomberg-Maitland M, Miller DP, Frost A, Frantz RP, Foreman AJ, Badesch DB, McGoon MD. The REVEAL Registry Risk Score Calculator in Patients Newly Diagnosed With Pulmonary Arterial Hypertension, Chest 2012;141:354–362. 21680644 10.1378/chest.11-0676

[18] Nasri A, Dupuis J, Carrier M, Racine N, Parent M, Ducharme A, Fortier A, Hausermann L, White M, Tremblay-Gravel M. Thirty-year trends and outcome of isolated versus combined group 2 pulmonary hypertension after cardiac transplantation, Front Cardiovasc Med 2022;9:841025. 36531737 10.3389/fcvm.2022.841025 PMC9755656

[19] Titz A, Mayer L, Appenzeller P, Müller J, Schneider SR, Tamm M, Darie AM, Guler SA, Aubert J, Lador F, Stricker H, Fellrath J, Pohle S, Lichtblau M, Ulrich S. Long-term outcome of patients with combined post- and pre-capillary pulmonary hypertension, Eur Heart J Open 2023;3:oead069. 37528902 10.1093/ehjopen/oead069 PMC10387509

[20] Senni M, Tribouilloy CM, Rodeheffer RJ, Jacobsen SJ, Evans JM, Bailey KR, Redfield MM. Congestive heart failure in the community: a study of all incident cases in Olmsted County, Minnesota, in 1991, Circulation 1998;98:2282–2289. 9826315 10.1161/01.cir.98.21.2282

[21] Lam CSP, Roger VL, Rodeheffer RJ, Borlaug BA, Enders FT, Redfield MM. Pulmonary hypertension in heart failure with preserved ejection fraction: a community-based study, J Am Coll Cardiol 2009;53:1119–1126. 19324256 10.1016/j.jacc.2008.11.051 PMC2736110

[22] Melenovsky V, Hwang S, Lin G, Redfield MM, Borlaug BA. Right heart dysfunction in heart failure with preserved ejection fraction, Eur Heart J 2014;35:3452–3462. 24875795 10.1093/eurheartj/ehu193 PMC4425842

